# Crystallization Morphology Regulation on Enhancing Heat Resistance of Polylactic Acid

**DOI:** 10.3390/polym12071563

**Published:** 2020-07-15

**Authors:** Yufei Liu, Siyuan Jiang, Wei Yan, Min He, Jun Qin, Shuhao Qin, Jie Yu

**Affiliations:** 1 Department of Polymer Material and Engineering, College of Materials and Metallurgy, Guizhou University, Guiyang 550025, China; gs.liuyf16@gzu.edu.cn (Y.L.); gs.syjiang18@gzu.edu.cn (S.J.); qi_njun@163.com (J.Q.); 2 National Engineering Research Center for Compounding and Modification of Polymeric Materials, Guiyang 550014, China; pec.shqin@gzu.edu.cn; 3 School of Chemistry and Materials, Guiyang University, Guiyang 550005, China; lrasyw@163.com

**Keywords:** polylactic acid, crystallization morphology, heat resistance

## Abstract

To expand the use of polylactic acid (PLA) in high-temperature environments, crystallization morphology regulation was studied to enhance the heat resistance of PLA. PLA crystallinity was controlled using heat treatment and nucleating agent (zinc phenylphosphonate, brand TMC). The heat deflection temperatures of PLAs with same crystallinities considerably varied using different treatments. The crystallization morphology of PLA (4032D) and PLA/TMC composites was studied using X-ray diffraction (XRD) and polarized optical microscopy. XRD test results show that TMC can improve the crystallization rate and heat treatment can enhance the crystallinity and thickness of PLA, suggesting that the crystallization morphology improved after heat treatment. Nucleating agents can increase the crystallinity of PLA but cannot improve its crystallization morphology. The findings indicate that at the same crystallinity, PLAs exhibit improved crystallization morphology and high heat resistance; these results can provide guidance for improving the heat resistance of PLAs and facilitate the design of new nucleating agents.

## 1. Introduction

Polylactic acid (PLA) has attracted considerable research interest owing to its good biocompatibility and biodegradability [1,2,3,4,5,6,7]. However, its disadvantages, such as poor heat-deflection temperature (HDT: 50 °C for neat PLA) and slow crystallization rate, limit its application [8,9,10,11,12,13]. HDT is a crucial physical quantity used to characterize the heat resistance of polymeric materials [14,15]. Therefore, extensive research has been conducted to improve the heat resistance of PLAs [16] (Table 1). In recent years, lignin (LG) [17], cellulose nanocrystals (CNCs) [18], glass fiber (GF) [19], corn fiber (CF) [20], D-lactide [21], short Kenaf fiber (KF) [22], and alloys [23,24,25,26,27,28,29] have been used to increase the HDT of PLAs through substantial research [30,31].

Using heat-treatment, Wang [32] studied the thermal resistance of GF-reinforced PLA composites and found that enhanced crystallization post heat treatment significantly improves the mechanical properties of neat PLA and PLA/GF composites. Pan [33] studied the HDT of PLA/short basalt fiber (SBF) composites and found that PLA/SBF composites comprising interfacial crystals exhibit a significantly higher overall HDT than neat PLA. Lin [34] increased the HDT of PLAs by incorporating polycarbonate (PC) into PLA and using annealing to accelerate the crystallization of the PLA phase; consequently, the increase in HDT was attributed to the formation of a rigid three-dimensional (3D) framework comprising rigid PC particles and PLA crystals in the PLA/PC blends. These studies indicate that the modification of PLA using fiber, PC or nucleating agents must undergo heat treatment to improve the HDT. Moreover, crystallinity improvement is a prerequisite for improving the HDT of PLA. However, these studies did not consider the effect of crystal morphology on thermal stability. Our recent research shows that thermal stability is not only associated with the crystallinity of PLA, but also to its crystallization morphology.

In this study, a set of experiments were conducted to study the impact of a nucleating agent and heat treatment on improving the crystallinity, crystallization morphology and HDT of PLA. The crystallization morphology regulation for enhancing the heat resistance of PLA was analyzed using differential scanning calorimetry (DSC), X-ray diffraction (XRD) characterization and polarized optical microscopy (POM). Our work indicates that at the same crystallinity, a more complete crystallization morphology corresponds to a better heat resistance of PLA; these findings provide guiding significance for improving the heat resistance of PLA and designing a new nucleating agent.

## 2. Experimental section

### 2.1. Materials and Sample Preparation

**Figure 1:**
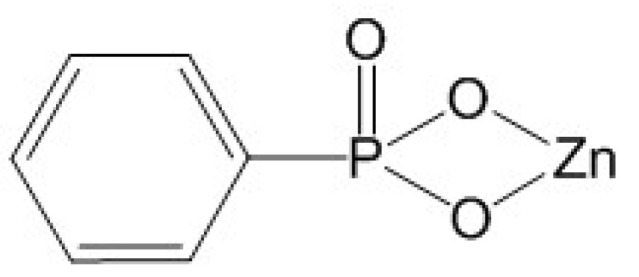
Chemical structure of zinc phenylphosphonate (brand TMC).

An extrusion molding grade of PLA, 4032D, was supplied by Natureworks (Blair, NE, USA). PLA has a density of 1.24 g cm^−3^ and glass transition temperature (*T*g) of 60 °C. The average molecular weight (*M*_w_) and numerically average molecular weight (*M*_n_) of PLA is 100 kg/mol and 58 kg mol^−1^, respectively. Commercial zinc phenylphosphonate (brand TMC) (Figure 1), a white powder, was purchased from Shanxi Chemical Research Institute Co., Ltd. (Jinzhong, Shanxi, China), and it has a melting point of 300 °C.

Both PLA and TMC were dried at 80 °C for 24 h in a vacuum oven. PLA in pellet form and white powder TMC (0.3 wt% and 0.5 wt%, respectively) were compounded using a twin-screw extruder (LHFD1-130718, Lab Tech Engineering Company, Ltd. Samutprakarnc, Thailand) with a screw diameter of 16 mm and length-to-diameter ratio of 40. The temperature interval in various zones from the hopper to die was set at 5 °C in the range of 165 °C–210 °C. The rotational speed of the screw was set at 100 rpm. Post extrusion, the strands were cooled with cold water and granulated with a granulator thereafter.

After blending, the PLA and PLA/TMC materials were again dried at 80 °C for 12 h to remove any residual moisture. Thereafter, the blends were injected into standard splines on an injection molding machine (PL860/290v, Wuxi Haitian Machinery Co. LTD, Jiangsu, China) at 210 °C for HDT, XRD, DSC and POM testing. The injection speed, packing pressure and packing time were set as 55 cm^3^/s, 5.0 MPa and 20.0 s, respectively.

To obtain samples with different crystallinities, the PLA (4032D) samples were treated at 110 °C for 20 and 30 min. The heat treatment durations of 20 and 30 min are deduced from our preliminary research.

### 2.2. Heat Deflection Temperature (HDT)

The effect of crystallinity on the HDT of PLA was determined using an HDT & VICAT testing machine (ZWK1000, MTS Systems (China) Co. LTD, Shenzhen, China). The measurements were performed according to GB/T1634.1–2004 with a constant load of 0.45 MPa and temperature rate of 120 °C h^−1^.

### 2.3. Bending Modulus

The bending modulus was tested according to GB/T9341-2008 with a bending rate of 2 mm min^−1^, fixture spacing of 80 mm, and using the average value of each group containing five samples.

### 2.4. Storage and Loss Moduli

Storage and loss modulus measurements were performed on a Q800 dynamic mechanical analysis instrument (DMA; TA Instruments, New Castle, DE, USA) at 1 Hz at a heating rate of 2 °C min^−1^ in a temperature range of 30 °C–120 °C. The high-temperature measurements were performed under a stream of dry N_2_.

### 2.5. Differential Scanning Calorimetric (DSC) Analysis

A Q20 DSC (TA Instruments, New Castle, DE, USA) was used to analyze the crystallization of heat-treated and untreated injection-molded PLA and PLA/TMC specimens. For a typical DSC test, a sample with a weight of approximately 2–4 mg was cut from the PLA specimen. Thereafter, the samples were heated to 210 °C at a rate of 10 °C min^−1^, held for 3 min to eliminate previous thermal history and cooled to 40 °C at a rate of 2 °C min^−1^ under N_2_ atmosphere. Based on the thermograms obtained from the first heating scan, the degree of crystallinity (*X*_c_) of the sample was calculated as follows:

(1) $$X_{c}=\Delta H_{m}/\left(\left(1-x\right)\Delta H_{m}^{0}\right)$$

where Δ*H*_m_ represents the crystallization enthalpy of the sample (J g^−1^); $\Delta H_{m}^{0}$ represents the enthalpy value of melting a 100% crystalline form of PLA, which is 93 J g^−1^; and x represents the weight fraction of TMC. To calculate the apparent activation energy of PLA and PLA/TMC (0.5 wt%), the samples were heated to 210 °C at a rate of 10 °C min^−1^, held for 3 min to eliminate previous thermal history and cooled to 40 °C at rates of 1 °C min^−1^, 2 °C min^−1^, 3 °C min^−1^ and 4 °C min^−1^ under N_2_ atmosphere.

Kissinger’s method, which considers the variation of the crystallization peak temperature (*T*_p_) with the cooling rate (φ), has been widely used to evaluate the overall effective energy barrier (Δ*E*). The Kissinger method is expressed as follows [35]:

(2) $$d(\ln(\varphi /T_{p}^{2}))/d(1/T_{p})=-\frac{\Delta E}{R}$$

where *R* (8.314) represents the gas constant. The slopes of the straight lines yield the value of Δ*E*/*R*.

### 2.6. X-ray Diffraction (XRD) Characterization

The influence of TMC and heat treatment on the crystal form of PLA was examined on the Rigaku (Ultima-IV) diffractometer equipped with a Cu–*K*α radiation source at a scan angle range of 5°–80° and scan rate of 1° min^−1^. Based on the XRD profiles, the crystallinity of PLA samples was calculated using the following formula [36]:

(3) $$X_{c}=I_{c}/(I_{c}+I_{a})$$

where *I*_c_ and *I*_a_ represent the integrated intensities scattered over a suitable angular interval by the crystalline and amorphous phases, respectively.

The dimensions of the crystals can be calculated using the following formula [37]:

(4) (D=(Kγ)/(Bcosθ)

where γ = 0.15406 is the X-ray wavelength, *K* is the Scherrer constant (*K* = 0.89), and B is the half-peak width of the XRD peak.

### 2.7. Polarized Optical Microscopy (POM)

The evolution of the crystal structure of the samples was observed using an Axio Scope A1 HC302 polarized optical microscope (Carl Zeiss, Jena, Germany) and heating stage (Instec, inc., Boulder, CO, USA) equipped with a digital camera. The samples were placed between two glass plates, melted at 200 °C on a Linkam THMS600 hot stage for 3 min to eliminate the thermal history and cooled to room temperature. Hereafter, portions of the samples were heat treated in a vacuum drying oven at 110 °C for 5 min.

## 3. Results and Discussions

### 3.1. Heat Deflection Temperature (HDT) and Moduli

**Figure 2:**
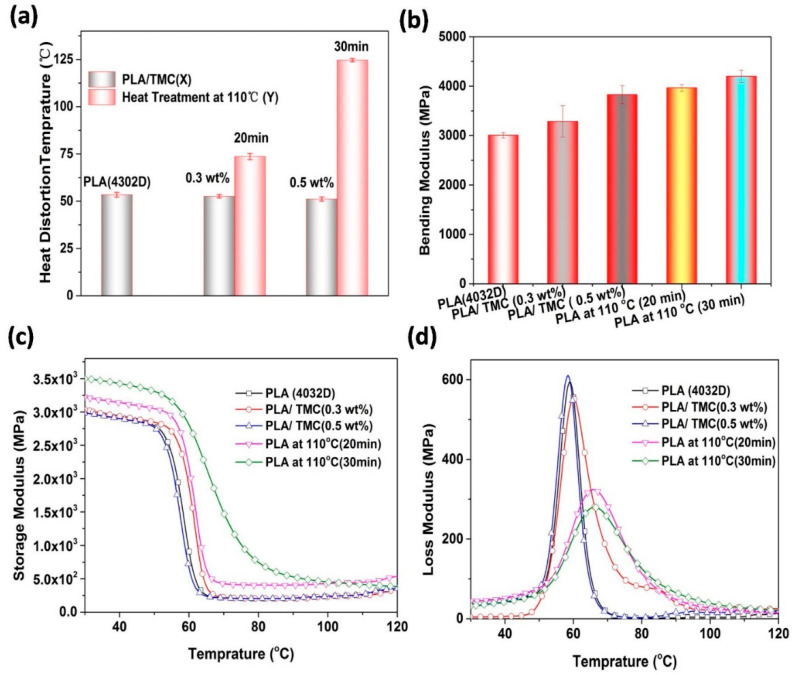
Heat-deflection temperature (HDT) and moduli of samples of neat PLA, PLA/TMC (0.3 wt% and 0.5 wt%) composites and PLA treated at 110 °C under varying heat treatment durations (20 and 30 min): (**a**) HDT, (**b**) bending modulus, (**c**) storage modulus, (**d**) loss modulus.

To evaluate the effects of the crystal morphology of PLA on its HDT, the HDT and moduli of PLA samples (treated at 110 °C under varying heat treatment durations of 20 and 30 min) and PLA/TMC (0.3 wt% and 0.5 wt%) composites were studied. As shown in Figure 2a, the samples with various amounts of TMC (0.3 wt% and 0.5 wt%) and no heat treatment show a similar HDT of approximately 50 °C, which indicates an insignificant effect of the increased crystallinity by TMC on the HDT of PLA. Conversely, post heat treatment at 110 °C for 20 and 30 min, both samples exhibited drastically improved HDT. The HDT of heat-treated PLA is consistent with that reported in the literature [38]. In particular, with nearly the same crystallinity (as observed in Figure 2a and Table 2), the HDT of PLA heat treated at 110 °C for 30 min was 124.6 °C, demonstrating an increase of 136.9% compared with the HDT of PLA with 0.5 wt% TMC. Clearly, the substantially improved HDT was attributed to not only the significantly enhanced crystallinity, but also the complete morphology of the crystal.

In HDT testing, a specific load is applied to the polymer material, after which the temperature is increased at a certain rate to reach the corresponding temperature where the specified deformation is reached. The test principles of HDT and the bending, storage and loss moduli have something in common, e.g., their test methods use the simply supported beam pressure. Therefore, the modulus of PLA samples was also examined for an in-depth analysis of HDT. Figure 2b–d shows that the bending and storage moduli of heat-treated PLA was higher than those of PLA/TMC composites and neat PLA. These findings are consistent with those obtained in Ikada’s report [39]. The loss modulus of heat-treated PLA was lower than that of PLA and PLA/TMC composites. The modulus test results show that the rigidity of heat-treated PLA was stronger than that of PLA and PLA/TMC composites [15]. The HDT results were verified through the results of bending and storage moduli. To illustrate the relationship between crystallization and HDT, the crystallinity, crystal type and crystal size of PLA were processed using different testing protocols: DSC, XRD and POM.

### 3.2. Differential Scanning Calorimetric (DSC)

**Figure 3:**
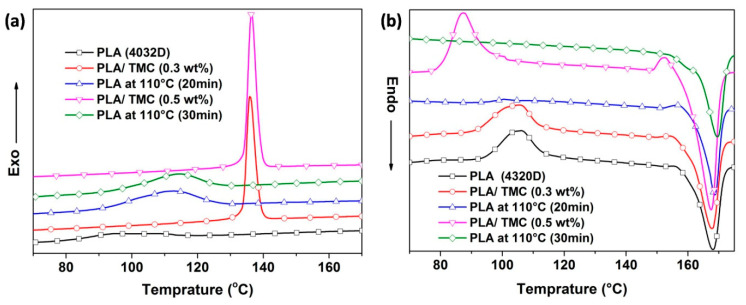
DSC curve of neat PLA, PLA/TMC (0.3 wt% and 0.5 wt%) composites and PLA (subjected to heat treatment at 110 °C for 20 and 30 min). (**a**) Crystallization and (**b**) melting curves.

The crystallinity behaviors of PLA and PLA/TMC composites were investigated by DSC analysis. Figure 3 shows the DSC thermograms of PLA post different heat treatments and PLA/TMC composites during the first heating stage. All DSC curves of PLA/TMC composites exhibited a cold crystallization peak and peak temperature of the crystallization of PLA. The peak temperature of crystallization of PLA is approximately 94.5 °C, which increased to 113.8 °C and 136.3 °C after adding TMC in PLA in 0.3 wt% and 0.5 wt% concentrations, respectively. Compared with neat PLA, the peak temperature of crystallization (113.8 °C and 136.3 °C) and crystallinity (21.3% and 30.1%) of PLA/TMC composites calculated using formula (1) gradually increased. These results are consistent with those obtained by Han [40] and Wua [41]. With increasing heat treatment durations, the crystallinity (21.3% and 30.1%) of PLA (subjected to heat treatment at 110 °C for 20 and 30 min) calculated using formula (1) in terms of thermodynamics gradually increased and the melting peak temperature remained unchanged. These data indicate that adding TMC and implementing heat treatment can improve the crystallinity of PLA. These experimental results are consistent with those obtained in Bai’s work [38].

**Figure 4:**
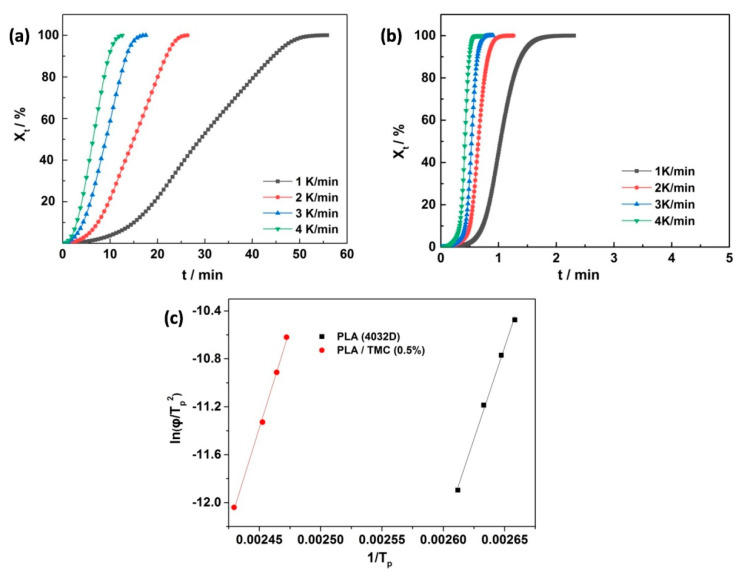
Crystallinity of (**a**) neat PLA and (**b**) PLA/TMC (0.5 wt%) versus crystallization time at varying crystallization rates; (**c**) Kissinger plots of ln (φ/T_p_^2^) for estimating crystallization activation energy.

Although PLA subjected to 30 min of heat treatment exhibited nearly the same crystallinity as PLA treated with 0.5 wt% TMC, their HDT values significantly differed. To investigate this difference, the crystallization activation energies of neat PLA and PLA/TMC (0.5 wt%) were studied (Figure 4).

Thus, the Δ*E* values of nonisothermal crystallization were −255 and −275 kJ mol^−1^ for neat PLA and PLA/TMC (0.5 wt%) composites, respectively. The absolute value of Δ*E* for PLA/TMC (0.5 wt%) was slightly higher than that of neat PLA, implying that the crystallization capacity of the PLA–MC composites was slower than that of neat PLA. However, the crystallinity of PLA/TMC (0.5 wt%) was higher than that of neat PLA. Therefore, TMC acted as a heterogeneous nucleating agent and did not change the energy barrier of the ordered arrangement of the PLA crystal molecular chains. These results were consistent with those obtained in Xu’s work [38].

### 3.3. X-ray Diffraction (XRD) Characterization

**Figure 5:**
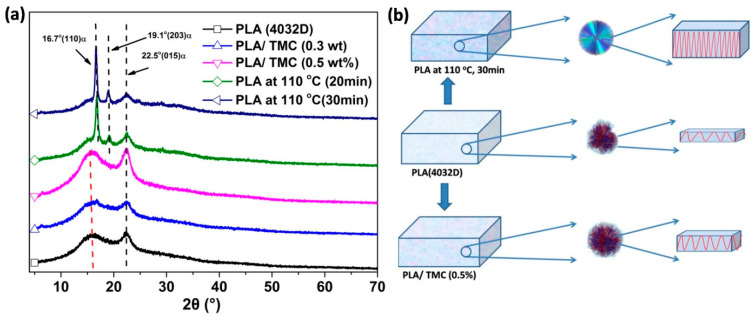
(**a**) X-ray diffractograms of neat PLA, PLA/TMC (0.3 and 0.5 wt%) and PLA (subject to heat treatment at 110 °C for 20 and 30 min); (**b**) schematic of the crystal morphology of neat PLA, PLA/TMC (0.5 wt%) and PLA (subject to heat treatment at 110 °C for 30 min).

Neat PLA, heat-treated PLA and PLA/TMC composites were independently analyzed using XRD, as shown in Figure 5a. All PLA/TMC composites exhibited diffuse diffraction peaks rather than sharp crystal diffraction peaks, indicating incomplete crystallization, which was inconsistent with DSC results. Moreover, PLA heat-treated for 20 and 30 min exhibited sharp crystal diffraction peaks at 2*θ* = 16.7°, 19.1° and 22.5°. These data indicate that the degree of crystallinity and reinforcement of PLA were influenced by heat treatment at varying times. According to formula 3, the crystallinity of PLA heat-treated at 110 °C for 20 and 30 min was 25.7% and 30.0%, respectively. These data indicate that the crystallinity increased as the heating time increased, which was consistent with the DSC result. According to formula 4, the size of the heat-treated PLA crystals was 1.2 and 2.5 nm, respectively. The results of the XRD and DSC experiments show that the addition of TMC could increase the crystallization rate of PLA; however, heat treatment improves the crystallinity of PLA, inducing a more complete PLA crystal. Therefore, a model for adding TMC and implementing heat treatment to improve PLA crystallization was proposed here, as shown in Figure 5b. Neat PLA exhibits low crystallinity and incomplete crystals. With the addition of TMC, more PLA crystals were formed, but the arrangement of the PLA molecular chains was not compact. Post heat treatment of neat PLA, the crystals were more prevalent, molecular chains were arranged more orderly, and thickness of the sheet crystals increased. To confirm our hypothesis, POM experiments were conducted.

### 3.4. Polarized Optical Microscopy (POM)

**Figure 6:**
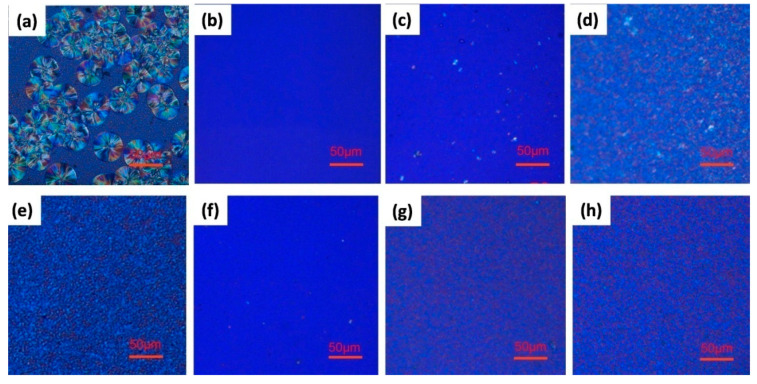
POM results of PLA and PLA/TMC (0.5 wt%): (**a**) PLA (4032D) heated at 130 °C for 30 min, (**b**) PLA (4032D) heated at 210 °C for 10 min, (**c**) PLA (4032D) heated at 30 °C from 210 °C at a rate of 10 °C min^−1^, (**d**) PLA (4032D) heated at 110 °C for 30 min from 30 °C at a rate of 10 °C min^−1^, (**e**) PLA/TMC (0.5 wt%) heated at 130 °C for 30 min, (**f**) PLA/TMC (0.5 wt%) heated at 210 °C for 10 min, (**g**) PLA/TMC (0.5 wt%) heated at 30 °C from 210 °C at a rate of 10 °C min^−1^ and (**h**) PLA/TMC (0.5 wt%) heated at 110 °C for 30 min from 30 °C at a rate of 10 °C min^−1^.

Figure 6a,e presents the POM micrographs of neat PLA and the PLA/TMC (0.5 wt%) composite post isothermal crystallization at 130 °C for 30 min. The observed neat PLA spherulites with a diameter of ~50 μm grew completely with the maltese cross. However, after the addition of TMC, the number of PLA spherulites increased and their size decreased. The spherulites became less complete, which may be attributed to spherulite interference. When the temperature of neat PLA decreased from 210 °C to 30 °C at a rate of 10 °C min^−1^, the spherulites of neat PLA were few in number, but large in size; these spherulites increased after 30 min of heat treatment. Conversely, the spherulites of PLA/TMC (0.5 wt%) the composite increased in number and decreased in size; further, the spherulites became complete after 30 min of heat treatment. The above results are consistent with the XRD analysis.

## 4. Conclusions

Conclusively, we optimized the crystallinity of PLA and increased the HDT of PLA by adding a nucleating agent and implementing heat treatment. The crystallinity of neat PLA was 11.7% and its HDT was 53.4 °C. The crystallinity of PLA treated at 110 °C for 30 min was nearly the same as that of PLA/TMC (0.5 wt%); however, the HDT of PLA treated at 110 °C for 30 min was 72 °C higher than that of PLA/TMC (0.5 wt%). The morphology of the PLA crystal was studied by XRD and POM. Results indicated that with the same crystallinity, the crystal obtained via heat treatment was more complete than the PLA/TMC (0.5 wt%) composite. Therefore, it was concluded that the crystallization morphology of PLA significantly affected its heat resistance. The findings of this study are therefore significant for examining the relationship between crystallinity, crystallization morphology and heat resistance.

## Figures and Tables

**Table 1:** Research progress on the heat deflection temperature (HDT) of polylactic acid (PLA).

PLA and Its Composites	HDT(°C)	Test Method	Heat Treatment	References
PLA	50	0.45 MPa	No	[16]
PLA/LG (20%)	54	0.45 MPa	No	[17]
PLA/CNC (20%)	78	DMA	Yes, at 130 °C	[18]
PLA/GF (40%)	55	1.82 MPa	No	[19]
PLA/GF (20%)	150	0.45 MPa	Yes, at 100 °C	[32]
PLA/CF (10%)	60	1.82 MPa	No	[20]
PLA/KF (40%)	135	0.45 MPa	Yes, at 80 °C	[22]
PLA/D-lactide (0.5 wt%)	150	0.45 MPa	Yes, at 140 °C	[21]
PLA/PHA (90/10)PLA/PHA	62	1.82 MPa	Yes, at 100 °C	[24]
	50	1.82 MPa	No	[24]
PLA/PP (60/40)	60	0.45 MPa	No	[26]
PLA/POM (40/60)	140	0.46 MPa	No	[29]

**Table 2:** Differential scanning calorimetric (DSC) parameters and HDT of PLA samples.

Samples	*X*_n_^a^ (%)	*T*_0_^b^ (°C)	*T*_p_^c^ (°C)	ΔH ^d^ (J/g)	*T*_m_^e^ (°C)	*HDT* (°C)
PLA (4032D)	11.7	113.7	94.5	10.9	168.0	53.4
PLA/TMC (0.3 wt%)	21.3	126.1	113.8	15.8	167.6	51.1
PLA at 110 °C (20 min)	25.3	126.7	113.3	47.9	169.7	73.6
PLA/TMC (0.5 wt%)	30.1	139.1	136.3	28.02	167.5	52.6
PLA at 110 °C (30 min)	30.84	139.0	135.9	43.3	167.7	124.6

^a^ crystallinity; ^b^ onset crystallization temperature; ^c^ peak temperature of crystallization; ^d^ melting enthalpy; ^e^ melting temperature of the first crystal.
